# Determination of aging anxiety in middle-aged women

**DOI:** 10.1590/1806-9282.20231220

**Published:** 2024-04-22

**Authors:** Nese Kiskac, Mahruk Rashidi, Muharrem Kiskac

**Affiliations:** 1 Istanbul Gelisim University, Faculty of Health Sciences, Department of Nursing – İstanbul, Turkey.; 2 Bezmiâlem Vakif University, Faculty of Medicine Hospital, Department of Internal Medicine – İstanbul, Turkey.

**Keywords:** Aging, Anxiety, Female, Gender

## Abstract

**OBJECTIVE:**

The aim of this study was to determine the state of aging anxiety in middle-aged women.

**METHODS:**

The study was collected from women between the ages of 40 and 59 years by an online survey method. While collecting the data of the participants, the women's personal characteristics diagnostic form and the Aging Anxiety Scale for Middle-Aged Women were used. The data were analyzed with the SPSS 26 statistical software.

**RESULTS:**

The aging anxiety of the women was found to be moderate (53.05±16.26). A significant correlation was found between women's menopausal status, household income, education level, and total score of aging anxiety (p<0.05).

**CONCLUSION:**

In addition to working outside the home, women are also burdened with duties inside the home. To improve their quality of life, women need to share many of the tasks imposed on them with other family members. To reduce the anxiety experienced by women during the climacteric period, it is recommended to provide psychosocial support to women and consider this issue in health policies. Healthcare professionals, especially nurses, have important duties to reduce anxiety and stress, which constitute the basis of many chronic diseases. It is recommended that nurses, who are health ambassadors, direct women with anxiety to psychological support services through screenings they will conduct for women during this period.

## INTRODUCTION

Aging is a process that has bio-psychosocial dimensions and cannot be prevented^1^. It is not enough to consider aging only chronologically. Many factors, such as environmental factors, lifestyle, genetic characteristics, and social and cultural life, affect aging. Therefore, it is necessary to address the concept of aging in all aspects^2^.

In recent years, the world's population has been aging rapidly^3^. The World Health Organization (WHO) reported that by 2030, one out of every six people will be over the age of 60 years, and by 2050, the world's population aged 60 years and over will be twofold^4^.

Gender plays an important role in determining the frequency of anxiety, onset time, course, and prognosis of diseases. In addition to biological and psychological predispositions, gender-specific social risk factors are also effective in the emergence of anxiety^5^. Factors such as women's position in society, conflicting work, family, and social roles, especially the burden of caring for children and family members, the low status of women, which is more prominent in certain societies, not having equal conditions in working life, and being poorer cause women to be more sensitive to stress. In addition, the fact that women enter the workforce today and carry out all their other roles while working at the same time increases the burden on them even more. Low employment and pension rights, more economic problems in older women, less participation in social environments, and more mental problems increase the fear of aging in middle-aged women^6^. According to the WHO, 45–59 years is defined as middle age^7^. These problems will increase even more with aging^8^.

Since the negative conditions of being a woman will make the living conditions more difficult in old age, it will increase the aging anxiety in middle-aged women^9^. According to studies, it has been determined that women between the ages of 40 and 65 years, which is defined as the climacteric period, experience a lot of anxiety^10^. When the literature is examined, it is seen that there are a limited number of studies in which the aging concerns of middle-aged women are measured and the conditions affecting their quality of life are evaluated^11-14^. When the literature studies are examined, it is seen that women in the climacteric period face many situations that will negatively affect their quality of life. The aim of this study was to determine the aging concerns of women aged 40–59 years. It is thought that the results of the research will shed light on the creation of health policies to be created for solutions in the future.

## METHODS

### Study design

The research is a descriptive and cross-sectional study.

### Sample of the research

The sample consisted of 240 women aged between 40 and 59 who agreed to participate in the study.

### Data collection method

The data for the study were collected from the participants in the form of an online survey between May 1 and June 30, 2023.

### Data collection

The data for the study were obtained by using the personal data identification form (age, marital status, education level, occupation, child status, with whom they live at home, menopausal status, and household income level) and the Aging Anxiety Scale for Middle-Aged Women (AASMAW).

### Aging anxiety scale for middle-aged women

The validity and reliability of the scale developed by Lee et al.^15^ was conducted by Daşıkan et al.^16^ The scale sample consisted of women aged 40–59 years. While language, content, and construct validity analyses were performed in the validity phase of the scale, internal consistency and stability over time analyses were performed in the reliability phase. Cronbach's α reliability is 0.962 for the overall scale and ranges between 0.836 and 0.949 for the four sub-dimensions. The Cronbach's α value of this study was 0.789. The scale consists of 19 items, and a 5-point Likert scale is used. The scale consists of four sub-dimensions: physical competence (questions 1–4), anxiety about changes in appearance (questions 5–8), social value (questions 9–16), and negative expectations about aging (questions 17–19). Questions 17–19 are reverse-scored. The total value of all sub-dimensions is calculated as a total score. As the total score increases, the level of aging anxiety also increases.

### Data analyses

The IBM SPSS 26.0 statistical program was used in the data analysis of this study. In addition to descriptive statistical methods (mean, standard deviation, frequency, and percentage), the Student's t-test was used to compare normally distributed data, and the Mann-Whitney U test was used to compare non-normally distributed data. One-way analysis of variance and Kruskal-Wallis test were used to evaluate more than two normal and non-normally distributed variables, respectively. The results were evaluated at a 95% confidence interval and a p<0.05 significance level.

### Ethical considerations

Ethics committee approval was obtained from the Istanbul Gelişim University Ethics Committee Presidency with the decision dated April 19, 2023 and numbered 2023-04-141 to conduct the research. The participants to be included in the study were informed before the survey, and a consent form was signed.

## RESULTS

The personal data of the participants are shown in Table 1. It was found that 84.2% of the women were married, 60.4% were university graduates, 70.8% were employed, 45.4% had two children, 67.9% were not in menopause, 93.3% lived with their families, 69.2% had medium household income, and the mean age was 46.34±5.24 years (Table 1).

**Table 1:** Personal data of the participants (n=240).

		n	%
Age (years; average)		46.34±5.24	
Marital status			
	Single	38	15.8
	Married	202	84.2
Educational levels			
	Illiterate	3	1.3
	Literate	2	0.8
	Primary school graduate	18	7.5
	Secondary school graduate	13	5.4
	High school graduate	59	24.6
	University graduate	145	60.4
Profession			
	Employee	170	70.8
	Pensioner	13	5.4
	Housewife	57	23.8
Child status			
	None	28	11.7
	1 child	58	24.2
	2 children	109	45.4
	3 children	28	11.7
	4 and more children	17	7.1
Menopausal status			
	There is	77	32.1
	None	163	67.9
Whom she lives with at home			
	Alone	14	5.8
	With her family	224	93.3
	With a friend	2	0.8
Household income level			
	Inadequate	23	9.6
	Medium	166	69.2
	Good	51	21.3

Descriptive statistical methods (mean, standard deviation, frequency, and percentage).

Table 2 shows the mean scores of the participants on the AASMAW and the relationship between them and their personal characteristics. The total score of the AASMAW was found to be 53.05±16.26 at a moderate level. A significant correlation was found between the menopausal status of the women and physical competence, the social value sub-dimension, and the total score of the AASMAW (p<0.05) (Table 2).

**Table 2:** The relationship between the personal characteristics of the participants and the sub-dimension and total scores of the Aging Anxiety Scale for Middle-Aged Women.

		Physical competence	p-value	Anxiety about changes in appearance	p-value	Social value	p-value	Negative expectations about aging	p-value	AASMAW total score	p-value
Average		12.66±4.28		10.84±4.71		20.98±8.58		8.58±3.33		53.05±16.26	
Menopausal status											
	There is	13.57±4.80	**0.02**	11.27±4.57	0.33	22.86±8.74	**0.02**	9.08±2.91	0.**10**	56.77±16.56	**0.01**
	None	12.23±3.95		10.64±4.77		20.09±8.39		8.34±3.49		51.29±15.86	
Age (years)											
	≥46	12.70±4.55	0.88	11.01±4.61	0.60	21.40±8.89	0.46	8.63±3.10	0.80	53.74±16.59	0.53
	<46	12.62±4.04		10.69±4.80		20.60±8.32		8.52±3.53		52.42±15.99	
Marital status											
	Married	12.76±4.22	0.41	10.83±4.82	0.91	21.00±8.51	0.94	8.67±3.34	0.31	53.24±16.39	0.67
	Single	12.13±4.63		10.92±4.08		20.89±9.10		8.08±3.26		52.02±15.71	

Statistically significant values are denoted in bold. Student's t-test, Mann-Whitney U test.

The relationship between the personal characteristics of the participants and the sub-dimensions of the AASMAW is shown in Table 3. A significant correlation was found between the participants’ education level and physical competence, social value, and AASMAW (p<0.05). It was observed that the anxiety of illiterates was higher than the others. A significant relationship was found between child status and physical competence (p<0.05). A significant correlation was found between the income level of the household and physical competence, social value, and AASMAW (p<0.05). Those who found the income level of the household insufficient were found to have higher anxiety than the others.

**Table 3:** The relationship between the personal characteristics of the participants and the sub-dimension and total scores of the Aging Anxiety Scale for Middle-Aged Women.

	Physical competence p-value	Anxiety about changes in appearance p-value	Social value p-value	Negative expectations about aging p-value	AASMAW total score p-value
Educational level	**0.009**	0.406	**0.002**	0.27	**0.004**
Child status	**0.036**	0.943	0.302	0.052	0.546
Whom she lives with at home	0.844	0.647	0.143	0.644	0.553
Household income level	**0.001**	0.924	**0.042**	0.244	**0.026**
Profession	0.930	0.134	0.240	0.214	0.518

Statistically significant values are denoted in bold. One-way analysis of variance, Kruskal-Wallis test.

## DISCUSSION

In this study, according to the results of the online questionnaire administered to 240 women aged 40–59 years, women's anxiety about aging was found to be moderate (53.05±16.26) (Table 2). The relationship between the personal characteristics of the participants and the score of the AASMAW was analyzed (Tables 2 and 3). There was a significant correlation between women's menopausal status, household income level, education level, and the AASMAW (p<0.05).

When the literature was analyzed, no study was found to have been conducted using the AASMAW. However, it is seen that there are a limited number of studies in which gender differences, age, occupation, education, socioeconomic factors, and anxiety are studied. Kiely et al.^17^ and Kessler et al.^18^ stated in their studies that women experience more anxiety about aging than men. Lytle et al.^19^ found that anxiety increased with age in a study conducted with 821 participants aged 45–80 years. Calatayud et al.^20^ found that anxiety was higher in those with low education. Carrard et al.^21^ conducted a study with 331 women aged 45–65 years and found that anxiety increased as women's physical body dissatisfaction increased and they used antiaging products. Woods et al.^22^ stated that menopause triggers memory disorders in women, and Castellon et al.^23^ stated that psychiatric problems associated with depression increase as memory disorders increase.

Kiely et al.^17^ and Kessler et al.^18^ stated in their studies that women experience more aging anxiety than men. The study was conducted with only females as a sample. However, the results of the literature reveal the purpose of this study. The fact that women are in a more disadvantaged group than men in factors such as hormonal, work, status, and economic conditions suggests that scientific studies to be conducted in the female gender should be increased.

Lytle et al.^19^ found that anxiety increased with increasing age in a study conducted with 821 participants aged 45–80 years. In the present study, the relationship between age and the AASMAW was not significant (p>0.05). The results of the literature study and this study are different. In the study, the sample was taken between 40 and 59 years old, while in the study of Lytle et al.^19^, the sample was taken between 45 and 80 years old. The reason for the difference in the results can be attributed to the difference between ages.

Calatayud et al.^20^ found that anxiety was higher in those with low education. In this study, a significant correlation was found between the level of education of the participants and physical competence, social value, and AASMAW (p<0.05). It was observed that the anxiety of illiterates was higher than the others. The result of the study is similar to the result of the literature. The fact that women with higher education level participate in business life and therefore have better social, retirement, and financial opportunities can be considered a factor that reduces anxiety.

Woods et al.^22^ stated that menopause triggers memory disorders in women, and Castellon et al.^23^ stated that psychiatric problems related to depression increased as memory disorders increased. In the present study, a significant correlation was found between menopausal status in women and the AASMAW (p<0.05). Those who had menopause had a higher level of aging anxiety. The result of this study is similar to that of the literature. We can explain the reason for this in two ways. First, in line with the literature, we can explain the decrease in memory functions with menopause and thus the increase in susceptibility to depression. Second, menopause is an inevitable process that usually occurs in women's advanced age. Women accept the hormonal changes in menopause and try to get used to the idea that they are getting older. We can say that this stage of acceptance affects the woman more emotionally and increases anxiety.

As a result of the review conducted by Aki^24^, it was stated that women's economic inadequacies increase depression even more. A significant correlation was found between the income level of the household and physical competence, social value, and AASMAW (p<0.05). In the study, those who stated that their household income was inadequate had a higher score than the others. The results of the study are similar to those in the literature. Participation of individuals in working life, being financially independent, and having an economic income in the retirement period are effective in accessing health, protecting health, and maintaining treatment and rehabilitation. In our country, men have more economic independence than women. We can say that women who think that their economic income is insufficient may have high aging anxiety because they think that their health will be negatively affected.

## CONCLUSION

In line with the literature data, this study was conducted considering that women's anxiety would be high because they are different from men both biologically and their status in society. As a result of the study, the aging anxiety score of women was found to be moderate. It was determined that women's menopause, low economic income, and low education levels increased the anxiety of aging. In addition to working outside the home, women are also burdened with duties inside the home. To improve their quality of life, women need to share many of the tasks imposed on them with other family members. To reduce the anxiety experienced by women during the climacteric period, it is recommended to provide psychosocial support to women and consider this issue in health policies. Healthcare professionals, especially nurses, have important duties to reduce anxiety and stress, which constitute the basis of many chronic diseases. It is recommended that nurses, who are health ambassadors, direct women with anxiety to psychological support services through screenings they will conduct for women during this period.
